# Supplementary material for: Artificial and natural selection components reveal the mechanisms of tropical sheep populations against gastrointestinal parasites
Source: PLoS One. 2026 Feb 18;21(2):e0340970. doi: 10.1371/journal.pone.0340970 (PMC12915954; doi:10.1371/journal.pone.0340970)
Supplement: S1 Table — The gene content and quantitative trait loci (QTL) associations are described for each genomic region, with QTLs classified as either health-associated traits (Health associated QTL) or other types according to the Animal QTL Database (Other associated QTL). (DOCX) [file pone.0340970.s001.docx]

| **Chromosome** | **Start** | **End** | **Selection component** | **Selection type** | **Overlay with ROH** | **Gene content** | **Health associated QTL** | **Other associated QTL** |
| --- | --- | --- | --- | --- | --- | --- | --- | --- |
| **1** | 120394997 | 123217898 | Natural | Stabilizing/disruptive | Yes | *ATF6, OLFML2B, NOS1AP, SPATA46, SH2D1B, UAP1, UHMK1, DDR2, HSD17B7, CCDC190, RGS4, RGS5, NUF2* | FECZ, FECGEN | MDLUMB3, BFLUMB3, FATP, FAC-C20:5, FA-C22:5, PUFA, BDENS, HORNT |
| **1** | 149085328 | 149585328 | Natural | Stabilizing/disruptive | No | *TMPRSS15* | NA | FATP, FA-C20:5, FA-C22:5, PUFA |
| **2** | 114465040 | 118245391 | Artificial | Stabilizing/disruptive | Yes | *GLRA3, CEP44, FBXO8, HAND2, SCRG1, SAP30, GALNT6, GALNT7* | MCH, NFEC, LATRICH_2 | UPH, FA-C18:3, FA-C20:5, FA-C22:5, CIEL, CIELA, CIELB, MFLAV, MTEND |
| **2** | 114746188 | 118883007 | Artificial | Directional | Yes | *GLRA3, CEP44, FBXO8, HAND2, SCRG1, SAP30, GALNT6, GALNT7* | CREAT, MHC, NFEC, LATRICH_2 | UPH, FA-C18:3, FA-C20:5, FA-C22:5, CIEL, CIELA, CIELB |
| **3** | 134320295 | 137268040 | Natural | Directional | Yes | *POC1B, DUSP6, ATP2B1, CCER1, LUM, EPYC, DCN, KERA* | DHCT, MVVS, CEOSIN | MCLA, BW, TNUM |
| **5** | 30065048 | 30565048 | Natural | Stabilizing/disruptive | No | *SNX2, PPIC* | NA | FATP, STA |
| **5** | 46276100 | 48818355 | Natural | Stabilizing/disruptive | Yes | *ABHD17A, SCAMP4, ADAT3, CSNK1G2, BTBD2, SHROOM1, ZCCHC10, HSPA4, FSTL4, GDF9, UQCRQ, LEAP2, AFF4, VDAC1, TCF7, CDKL3, UBE2B, JADE2, CDKN2A, IPNL, SAR1B, SEC24A, CAMLG, DDX46, PCBD2, DDX46, CATSPER3, PITX1, TXNDC15* | NA | BW, ATLOC, FOOTANG |
| **5** | 98658014 | 103093651 | Natural | Directional | Yes | *NR2F1, FAM172A, KIAA0825, SLF1, MCTP1, FAM81B, TTC37, ARSK, GPR5150, ELL2, SPATA9, GLRX, PCSK1, CAST, ERAP1, ERAP2, LNPEP, LIX1, RIOK2* | HCT | BW |
| **9** | 22681901 | 23181901 | Natural | Stabilizing/disruptive | Yes | *NDRG1, TG, SLA, ST3GAL1* | SAOS | MUSWT, HCWT, LMA |
| **9** | 85054852 | 85554852 | Natural | Stabilizing/disruptive | No | *VPS13B, RGS22* | NA | MUSWT, HCWT, LMA |
| **10** | 23932516 | 24432516 | Natural | Directional | No | *SLC25A15, MRPS31, FOXO1, VPS36, THSD1* | IGA, FECGEN | FATWT, FATP, HO, BONEP, LMYP |
| **12** | 27073654 | 29818542 | Natural | Directional | Yes | *DUSP10, HHIPL2, AIDA, BROX, FAM177B, DISP1, SUSD4, CCDC185, CAPN2, CAPN8, TP53BP2, FBXO28, DEGS1* | NA | LMYP, FATP, BDENS |
| **12** | 33519660 | 36068031 | Natural | Directional | Yes | *SMYD3, KIF26B, EFCAB2, COX20, HNRNPU, DESI2, ADSS2* | NA | LMYP, FATP, BDENS, REACT |
| **13** | 16202545 | 18555214 | Artificial | Directional | Yes | *CELF2, USP6NL, ECHDC3, UPF2, DHTKD1, SEC61A2, NUDT5, CDC123, CAMK1D, PRR13, CCDC3, YME1L1, MASTL, ABI1, PDSS1, APBB1IP, CCNY, CUL2, CREM* | ENTR, SAOS | SPLWT, MUSWT, CTVOC |
| **16** | 71666406 | 72166406 | Artificial | Directional | No | *ADCY2* | NA | LMYP, DRESSING |
