# Supplementary material for: Artificial and natural selection components reveal the mechanisms of tropical sheep populations against gastrointestinal parasites
Source: PLoS One. 2026 Feb 18;21(2):e0340970. doi: 10.1371/journal.pone.0340970 (PMC12915954; doi:10.1371/journal.pone.0340970)
Supplement: S2 Table — (DOCX) [file pone.0340970.s002.docx]

| Selection component | KEGG pathway |
| --- | --- |
| Artificial | oas00280: Valine, leucine and isoleucine degradation |
|  | oas00410: beta-Alanine metabolism |
|  | oas00640: Propanoate metabolism |
|  | oas00830: Retinol metabolism |
|  | oas01200: Carbon metabolism |
|  | oas03008: Ribosome biogenesis in eukaryotes |
|  | oas03015: mRNA surveillance pathway |
|  | oas03020: RNA polymerase |
|  | oas03022: Basal transcription factors |
|  | oas03420: Nucleotide excision repair |
|  | oas04066: HIF-1 signaling pathway |
|  | oas04080: Neuroactive ligand-receptor interaction |
|  | oas04666: Fc gamma R-mediated phagocytosis |
|  | oas05211: Renal cell carcinoma |
|  | oas05214: Glioma |
| Natural | oas00230: Purine metabolism |
|  | oas00240: Pyrimidine metabolism |
|  | oas00310: Lysine degradation |
|  | oas00330: Arginine and proline metabolism |
|  | oas00512: Mucin type O-glycan biosynthesis |
|  | oas00514: Other types of O-glycan biosynthesis |
|  | oas00600: Sphingolipid metabolism |
|  | oas00760: Nicotinate and nicotinamide metabolism |
|  | oas00983: Drug metabolism - other enzymes |
|  | oas01230: Biosynthesis of amino acids |
|  | oas01232: Nucleotide metabolism |
|  | oas01240: Biosynthesis of cofactors |
|  | oas01521: EGFR tyrosine kinase inhibitor resistance |
|  | oas01522: Endocrine resistance |
|  | oas03013: Nucleocytoplasmic transport |
|  | oas03018: RNA degradation |
|  | oas03040: Spliceosome |
|  | oas03082: ATP-dependent chromatin remodeling |
|  | oas03083: Polycomb repressive complex |
|  | oas03410: Base excision repair |
|  | oas04012: ErbB signaling pathway |
|  | oas04014: Ras signaling pathway |
|  | oas04022: cGMP-PKG signaling pathway |
|  | oas04024: cAMP signaling pathway |
|  | oas04061: Viral protein interaction with cytokine and cytokine receptor |
|  | oas04064: NF-kappa B signaling pathway |
|  | oas04068: FoxO signaling pathway |
|  | oas04070: Phosphatidylinositol signaling system |
|  | oas04071: Sphingolipid signaling pathway |
|  | oas04072: Phospholipase D signaling pathway |
|  | oas04110: Cell cycle |
|  | oas04142: Lysosome |
|  | oas04145: Phagosome |
|  | oas04146: Peroxisome |
|  | oas04150: mTOR signaling pathway |
|  | oas04151: PI3K-Akt signaling pathway |
|  | oas04152: AMPK signaling pathway |
|  | oas04210: Apoptosis |
|  | oas04211: Longevity regulating pathway |
|  | oas04213: Longevity regulating pathway - multiple species |
|  | oas04217: Necroptosis |
|  | oas04218: Cellular senescence |
|  | oas04270: Vascular smooth muscle contraction |
|  | oas04310: Wnt signaling pathway |
|  | oas04330: Notch signaling pathway |
|  | oas04350: TGF-beta signaling pathway |
|  | oas04380: Osteoclast differentiation |
|  | oas04514: Cell adhesion molecules |
|  | oas04550: Signaling pathways regulating pluripotency of stem cells |
|  | oas04610: Complement and coagulation cascades |
|  | oas04613: Neutrophil extracellular trap formation |
|  | oas04620: Toll-like receptor signaling pathway |
|  | oas04621: NOD-like receptor signaling pathway |
|  | oas04622: RIG-I-like receptor signaling pathway |
|  | oas04623: Cytosolic DNA-sensing pathway |
|  | oas04630: JAK-STAT signaling pathway |
|  | oas04650: Natural killer cell mediated cytotoxicity |
|  | oas04668: TNF signaling pathway |
|  | oas04670: Leukocyte transendothelial migration |
|  | oas04710: Circadian rhythm |
|  | oas04713: Circadian entrainment |
|  | oas04722: Neurotrophin signaling pathway |
|  | oas04723: Retrograde endocannabinoid signaling |
|  | oas04724: Glutamatergic synapse |
|  | oas04725: Cholinergic synapse |
|  | oas04727: GABAergic synapse |
|  | oas04742: Taste transduction |
|  | oas04750: Inflammatory mediator regulation of TRP channels |
|  | oas04810: Regulation of actin cytoskeleton |
|  | oas04910: Insulin signaling pathway |
|  | oas04911: Insulin secretion |
|  | oas04913: Ovarian steroidogenesis |
|  | oas04914: Progesterone-mediated oocyte maturation |
|  | oas04915: Estrogen signaling pathway |
|  | oas04916: Melanogenesis |
|  | oas04918: Thyroid hormone synthesis |
|  | oas04919: Thyroid hormone signaling pathway |
|  | oas04920: Adipocytokine signaling pathway |
|  | oas04922: Glucagon signaling pathway |
|  | oas04923: Regulation of lipolysis in adipocytes |
|  | oas04924: Renin secretion |
|  | oas04926: Relaxin signaling pathway |
|  | oas04927: Cortisol synthesis and secretion |
|  | oas04928: Parathyroid hormone synthesis, secretion and action |
|  | oas04929: GnRH secretion |
|  | oas04931: Insulin resistance |
|  | oas04933: AGE-RAGE signaling pathway in diabetic complications |
|  | oas04934: Cushing syndrome |
|  | oas04935: Growth hormone synthesis, secretion and action |
|  | oas04936: Alcoholic liver disease |
|  | oas04960: Aldosterone-regulated sodium reabsorption |
|  | oas04961: Endocrine and other factor-regulated calcium reabsorption |
|  | oas04962: Vasopressin-regulated water reabsorption |
|  | oas04964: Proximal tubule bicarbonate reclamation |
|  | oas04970: Salivary secretion |
|  | oas04971: Gastric acid secretion |
|  | oas04972: Pancreatic secretion |
|  | oas04973: Carbohydrate digestion and absorption |
|  | oas04974: Protein digestion and absorption |
|  | oas04976: Bile secretion |
|  | oas04977: Vitamin digestion and absorption |
|  | oas05032: Morphine addiction |
|  | oas05034: Alcoholism |
|  | oas05100: Bacterial invasion of epithelial cells |
|  | oas05143: African trypanosomiasis |
|  | oas05144: Malaria |
|  | oas05145: Toxoplasmosis |
|  | oas05150: Staphylococcus aureus infection |
|  | oas05152: Tuberculosis |
|  | oas05160: Hepatitis C |
|  | oas05161: Hepatitis B |
|  | oas05162: Measles |
|  | oas05163: Human cytomegalovirus infection |
|  | oas05164: Influenza A |
|  | oas05166: Human T-cell leukemia virus 1 infection |
|  | oas05167: Kaposi sarcoma-associated herpesvirus infection |
|  | oas05168: Herpes simplex virus 1 infection |
|  | oas05169: Epstein-Barr virus infection |
|  | oas05170: Human immunodeficiency virus 1 infection |
|  | oas05202: Transcriptional misregulation in cancer |
|  | oas05205: Proteoglycans in cancer |
|  | oas05206: MicroRNAs in cancer |
|  | oas05207: Chemical carcinogenesis - receptor activation |
|  | oas05213: Endometrial cancer |
|  | oas05217: Basal cell carcinoma |
|  | oas05224: Breast cancer |
|  | oas05225: Hepatocellular carcinoma |
|  | oas05226: Gastric cancer |
|  | oas05322: Systemic lupus erythematosus |
|  | oas05410: Hypertrophic cardiomyopathy |
|  | oas05412: Arrhythmogenic right ventricular cardiomyopathy |
|  | oas05414: Dilated cardiomyopathy |
|  | oas05417: Lipid and atherosclerosis |
|  | oas05418: Fluid shear stress and atherosclerosis |
| Both components | oas00190: Oxidative phosphorylation |
|  | oas00900: Terpenoid backbone biosynthesis |
|  | oas01100: Metabolic pathways |
|  | oas03010: Ribosome |
|  | oas04010: MAPK signaling pathway |
|  | oas04015: Rap1 signaling pathway |
|  | oas04020: Calcium signaling pathway |
|  | oas04060: Cytokine-cytokine receptor interaction, |
|  | oas04062: Chemokine signaling pathway |
|  | oas04070: Phosphatidylinositol signaling system |
|  | oas04120: Ubiquitin mediated proteolysis |
|  | oas04141: Protein processing in endoplasmic reticulum |
|  | oas04144: Endocytosis |
|  | oas04216: Ferroptosis |
|  | oas04260: Cardiac muscle contraction |
|  | oas04261: Adrenergic signaling in cardiomyocytes |
|  | oas04360: Axon guidance |
|  | oas04390: Hippo signaling pathway |
|  | oas04520: Adherens junction |
|  | oas04530: Tight junction |
|  | oas04540: Gap junction |
|  | oas04611: Platelet activation |
|  | oas04714: Thermogenesis |
|  | oas04814: Motor proteins |
|  | oas04912: GnRH signaling pathway |
|  | oas04921: Oxytocin signaling pathway |
|  | oas04925: Aldosterone synthesis and secretion |
|  | oas04932: Non-alcoholic fatty liver disease |
|  | oas05010: Alzheimer disease |
|  | oas05012: Parkinson disease |
|  | oas05014: Amyotrophic lateral sclerosis |
|  | oas05016: Huntington disease |
|  | oas05020: Prion disease |
|  | oas05132: Salmonella infection |
|  | oas05165: Human papillomavirus infection |
|  | oas05171: Coronavirus disease - COVID-19 |
|  | oas05200: Pathways in cancer |
|  | oas05208: Chemical carcinogenesis - reactive oxygen species |
