# Supplementary material for: Artificial and natural selection components reveal the mechanisms of tropical sheep populations against gastrointestinal parasites
Source: PLoS One. 2026 Feb 18;21(2):e0340970. doi: 10.1371/journal.pone.0340970 (PMC12915954; doi:10.1371/journal.pone.0340970)
Supplement: Attachment — Submitted filename: ResponseReviewers.pdf [file pone.0340970.s011.pdf]

AUTHOR'S REVIEW COMMENTS – REVIEWER 3

GENERAL COMMENTS

The reported infection levels appear unrealistically low and inconsistent. It seems implausible to accurately determine infection at such levels. Although the methodology states that FEC values were log_{10}-transformed, this is not reflected in the table, where values are still presented as eggs per gram. Furthermore, even if the data were log_{10}-transformed, average values such as 11 and 14 seem unusually high, suggesting a possible issue with either the examination or the transformation process. It is recommended to verify and, if necessary, repeat any analyses involving these values.

In fact, the data as presented is incorrect. For presentational reasons, we chose to preserve the raw data in the table, although the transformed data were used—correctly—in the analyses.

In reality, egg counts were performed on one-hundred-gram samples, and the data were later adjusted to one gram. In this process, a simple oversight led to a magnitude error. These are not low infections, even though infections varied significantly within farms and especially between farms. We apologize for the error.

The QTLs and genes discussed in the study appear to have been conducted from scan genomic regions identified by selection signature; however, this is not clearly explained in the methodology or results sections. Additionally, using a heading such as 'Quantitative Trait Loci Detection' in the results may be misleading, as the analysis does not involve a direct association with measured phenotypic traits.

Thank you for your insightful comment. We agree that the methodological explanation regarding the origin of QTL and gene annotations in the identified regions was not sufficiently clear. We have revised the Results section to clarify that the QTL data were retrieved from the Sheep QTLdb and intersected with the genomic regions identified through selection signature analyses. Additionally, we have changed the section heading from "Quantitative Trait Loci Detection" to "Annotation of selection regions with QTL data" to more accurately reflect the nature of the analysis, which did not involve direct phenotype-genotype associations.

Many fixed effects were included in the model, but there is no indication of which had a significant impact.

Mention of the contemporary group as to significant effects was added.

Some sections of the results are more likely to be considered as discussion which need to be revised and moved to the discussion section.

These topics were rewritten and the Discussion was subdivided to mark the discussion of these gene content results and the subsequent general discussion. We also merged the gene content topics into a single topic in the Results.

SPECIFIC COMMENTS

Introduction:
Page 4 line 4: "Haemonchus contortus Rudolph" what is Rudolph? It seems to be an author name
Rudolph is indeed the author's name. We removed this name and the others.

Results:
Throughout the results section, there is a recurring error in the abbreviation of Total Plasma Protein, which is incorrectly written as TTP. The correct abbreviation should be TPP
The text was rewritten with the change.

Page 8 line 17-18: "TTP and PCV showed the highest correlations overall, and using BCS, respectively, while FEC displayed the lowest correlations in both cases." Something missing in the sentence and TTP should be TPP
It was a syntax issue, and the text was rewritten. It was indeed confusing.

Materials and Methods:
Page 24 line 15: Details on the number of pedigree records and the number of generations should be provided.
Details have been included.

Page 24 line 23: It is unclear what permanent environmental effects were tested in the model.
Permanent environmental effects were the fixed effects. The text was changed to clarify this, including its initial breakdown of generic and residual effects.

Page 25 line 7-12: The number of levels or classes for each fixed effect should be clearly specified.
The levels were discrete and an attempt was made to clarify the issue in the text regarding the unexplained effects.

Page 25 line 9: "age class (1 = 0 days, 2 = 1 to 150 days, 3 = 151 to 550 days, 4 = more than 550 days)" It is illogical to use a class for age zero days. How were measurements taken at zero age.
These data were tested in the context of a larger project, including birth weight. However, for the current study, this class has now been removed.

Tables and figures:
The quality of the figures is poor and they need to be reproduced in higher resolution.
The figures were generated again.

Table 1: The numbers for PCV are not presented as %
The text was rewritten with the change.

Table 1 and 2: Total Plasma Protein (TTP) should be Total Plasma Protein (TPP) in the header and inside tables

The text was rewritten with the change.

Table 2: I suggest to include genetic correlation

The text was rewritten with the change.

Table 3: "CV%" should be PCV %

The text was rewritten with the change.

Table 3: Standard deviations or standard errors should be added to the reported averages. Additionally, an ANOVA analysis comparing group means is missing and should be conducted to determine if the differences between groups are statistically significant.
Table S2 is not included and the link produce Table S1

We understand that this was unclear. The difference between groups in parasitology was significant at the genetic level and in the parameters analyzed, as confirmed by the analysis of variance and STRUCTURE. To assess the difference between farms, since the selective analysis contrasted the farm with the highest challenge (highest level of infection) and the farm with the lowest challenge (lowest level of infection), the results of a mean comparison test (also cited in the text) have now been added to Table 1, showing that all four traits differed.
