# Supplementary material for: Artificial and natural selection components reveal the mechanisms of tropical sheep populations against gastrointestinal parasites
Source: PLoS One. 2026 Feb 18;21(2):e0340970. doi: 10.1371/journal.pone.0340970 (PMC12915954; doi:10.1371/journal.pone.0340970)
Supplement: Attachment — Submitted filename: Response_to_Reviewers.pdf [file pone.0340970.s012.pdf]

AUTHORS' REVIEW COMMENTS – REVIEWER 3

FIRST COMMENT

Due to editorial reasons related to copyright, Figure 1 was removed. Consequently, Figure 2 became Figure 1, and so on.
Figure 5 (in the new version, Figure 4) has been redone for the same reason.

GENERAL COMMENTS

The table headers should be placed directly above the tables, with no text in between. Any accompanying text or explanation should follow the table as a separate paragraph, beginning with a reference to the table or figure number. For example, table 1 and 2 page 7.
The titles have been redone and we believe we have followed the recommendation now.

Some paragraphs in the Results section and the beginning of the Discussion are too short and should be merged with the following paragraphs to improve flow and coherence.
The paragraphs have been adjusted

Table 3. FEC units should be shift to same units as table 1, and standard deviations should be added.
The table was rebuilt with the correct FEC unit and standard deviations added. In fact, the FEC data was transformed and not readily intelligible.

Figure 3 and 4. The text within the figure should be enlarged in font size to ensure readability.
Figure 3 and 4 have been redone (in the new version, Figures 2 and 3).
