# Supplementary material for: Artificial and natural selection components reveal the mechanisms of tropical sheep populations against gastrointestinal parasites
Source: PLoS One. 2026 Feb 18;21(2):e0340970. doi: 10.1371/journal.pone.0340970 (PMC12915954; doi:10.1371/journal.pone.0340970)
Supplement: Attachment — Submitted filename: ResponseReviewers.docx [file pone.0340970.s010.docx]

**REVIEW COMMENTS TO THE AUTHOR**

**# REVIEWER 1**

*This is an interesting manuscript that contains a lot of useful information. The sample size is reasonably large and comprehensively analysed. The results on the means, standard deviations and correlations are clear and well presented. The quantitative genetic analyses seem appropriate but it would be useful to present the heritabilities in a table. The QTL data are useful but not very well presented. It is not clear how the QTL were identified. Simply stating 'Of the 49 QTL identified ...' lacks sufficient detail on how they were identified. Similar 'Overlay analysis ...' is unclear without a description of what exactly was done. This section needs to be rewritten to include precise descriptions of what exactly was done.*

**The mean heritabilities were inserted in Table 2. The texts in the results and methodology were adjusted, but we do not know if they were sufficiently explained in the way the reviewer intended. The QTLs were not identified in an original way, but compared with a database. If this alternative is considered uninformative, we suggest a new review so that we can suppress the results of quantitative trait loci.**

*The main concern is the attempt to quantify natural and artificial selection. The methodology is not clear. Simply citing references and then saying we did it differently is unhelpful. The assumptions and hypotheses are unconvincing. The usual assumption is that there will be genetic correlations among the various traits. The sample size is insufficient to provide precise estimates of genetic correlations but estimates have been provided for other breeds. These genetic correlations could account for the selective improvements observed. Undoubtedly, there is potential for both natural and artificial selection for parasite resistance but trying to quantify their relative contribution with unclear methodology is unconvincing.*

**The methodology was better detailed. Regarding the contribution of each selection component (natural or artificial selection), a parallel was drawn with classic studies involving sexual selection in the 1980s.**

*I did not see Table S1.*

**We do not know why it was not available, but in the new submitted file it is possible to see.**

*There are problems with the way the information is presented starting with the titles of the authors. eg Postdoctoral Professor and postdoctora.*

**We seek to adapt.**

Should 'Cody condition score' in the abstract be 'Body condition score'?

**Yes. It was an unseen typo.**

*Page 4 Line 12 'genomic regions' better as 'conserved regions'.*

**Changed in text.**

*Page 5 Line 5 'relaxed natural selection' might be clearer as 'weak natural selection'. It is not clear how artificial selection can be undirected.*

**The text has been better clarified. We understand that selection can be weak or strong depending on the intensity of selection practiced, and this can vary not only by the degree of directional selection, but also by the correlation with other traits. Thus, relaxed natural selection is not only weak selection, but it is especially weak selection that used to be strong in the wild but is no longer so due to issues such as health care, diet, and shelter. In turn, artificial selection can be indirect when the trait has a high correlation with another trait, which is selected.**

**Thus, since there are no breeding programs in sheep for resistance to worms, such resistance is the product of either natural selection that was relaxed in a captive environment (weak selection that used to be strong) or selection for another trait that led to resistance by draft (indirect artificial selection).**

*Page 6 line 4. PCV, TTP, ECC and FEC are not immunological traits. Perhaps parasitological or pathophysiological?*

**Changed in text.**

*Page 6 The legend for Fig 1. is partly repeated in the M & M. The previous study should be cited.*

**Changed in text.**

*Page 6 Line 23. Do not say that the table includes the CV for all data when it doesn't.*

**Changed in text.**

*Page 8 'Production trait' not 'productive trait'; this has another meaning.*

**Changed in text.**

*Page 8 line 13 The 'combined analysis' needs more explanation.*

**Changed in text.**

*Page 9 line 4. How can genomic regions contain health traits? Please rephrase.*

**The nomenclature was not given by us, but by the program used as reference. We understand the disagreement and, as previously discussed, this issue can be readjusted.**

*Page 9 Line 8 'modelled functions' needs elaboration.*

**Changed in text.**

*Page 12 Line 20 'pathways to artificial selection cluster' to 'pathways in the artificial ...'.*

**Changed in text.**

*Page 13 Line 9 onwards. The Resistant Th2 and susceptible Th1 pathways use fatty acid and carbohydrate catabolism to provide energy.*

*The discussion on pathways identified by KEGG is long and belongs in the discussion. The attempt to link all the pathways to immune mechanisms is unconvincing.*

**Changed in text. The option to describe it this way was used to make the discussion more fluid. If it is still long, inadequate and/or unconvincing, we can make a new change, including restricting the scope of the results.**

*Page 20 Line 12. O-linked glycosylation of mucin proteins is clearer.*

**Changed in text.**

*Page 21 Line 10. Perhaps indicate why BCS is preferred to body weight as a marker trait.*

**Changed in text**

*Page 22 Line 19 Not clear how or why FEC was adjusted.*

**Changed in text.**

*Page 22 Line 20 Coprocultures to identify and count nematodes?*

**Coproculture was used to estimate the proportion of the nematode species. We changed the text to make it clearer.**

*Page 28 Line 3. How was the gene content estimated by Fst?*

**Once the regions were estimated by Fst, the gene content of these regions was investigated, with differences in the analysis windows due to overlap or not with a ROH. Please indicate if this is still not clear.**

**# REVIEWER 2**

*Reviewer #2: The study ‘Artificial and natural selection components reveal the mechanisms of tropical sheep populations against gastrointestinal parasites’ is based on the phenotyping of resistance to Haemonchus contortus in a population of 1283 Santa Inês sheep distributed across 5 farms in the south and southeast regions of Brazil. Research combining molecular (Ovine SNP50 Genotyping BeadChip Illumina) and repeated phenotypic data (BW, BCS, FEC, PCV, ECC, TTP) is conducted, with an evolutionary approach being a relevant strategy for understanding the genetic history of immunological traits, including conserved genomic regions such as islands of homozygosity and selection signatures : breeding values (BLUPF90 software family), intensity of natural and artificial selection (statistics models), selection signatures (Wright's fixation index, runs of homozygosity), genes and associated pathways (Ensembl BioMart tool and the Panther Classification System, QTL alignments), selection type analysis (Lande & Arnold and Schuler methods, Bayescan software). The variable infestation levels between these 5 populations are interpreted as a gradient of natural selection pressures.*

*The manuscript concludes that both types of selection contribute to the phenomena of resistance and resilience. However, evidence of directional selection, hard sweeps, and functional enrichment of innate immunity was found for artificial selection, while natural selection exhibited evidence of stabilizing selection, soft sweeps, and functional enrichment of adaptive immunity.*

**The overview of the article was the one intended by the authors.**

*This is an impressive work in terms of the quantity of animals phenotyped (but over 20 years) and of results produced, but not detailed enough in each of the methods. The phenotyping protocol, the phenotypes recorded, the animals genotyped and the number of animals are not clearly understood.*

**The authors understand the critical review and have tried to make the methodology clearer. We expect additional comments, if necessary, so that a new review can be carried out if the explanation is still considered insufficient.**

*- 1,283 sheep were phenotyped 3 to 6 times at a median interval of one month, for a total of 2,241 records!*

**In fact, there was a problem in counting records and this was corrected in the text (3,845 measurements). There were few animals with more than three measurements and the long study time is due to the differences between populations and the generations of animals in some of these populations.**

*- Is the pedigree recorded or reconstructed by the genomic matrix?*

**The pedigree was recorded by herdbooks and then aligned by the matrix. Information was added to the text.**

*- Infestation is a count corrected for the estimated rate of H. contortus by coproculture. Why was the multi-species infection rate not considered? Is the correction individual or based on a pool of faeces? Specify the proportion of H.contortus on average (plus minimum-maximum). Does this proportion have an impact on the QTL identified?*

**QTLs were not originally identified, but rather regions whose gene content was being explored by selection signature were investigated for QTLs by previously available studies. If such an approach seems unconvincing, we suggest suppressing the information related to QTLs.**

**The approach of using the *Haemonchus* sp. proportion is related to the history of damage in the tropical herds analyzed, however other genera were identified, such as *Trichostrongylus* sp., with the proportion added in the text.**

*- Table 1 add numbers*

*• PCV is not expressed in the correct unit, in fact in %.*

**Changed in text.**

*• Infection levels are surprisingly low and very comparable, or unit error?*

**Infections appear low because these are average data, but there is great variability between farms. There are populations that have shown high levels of deworming, although this issue was considered in the modeling.**

*- Specify the rearing practices that generate these differences in infection levels between farms 1 and 5.*

**Changed in text.**

*- Table 2 specify pearson correlation*

**Added information in the methodology.**

*- Is the ECC score a compromise between the 2 evaluators or the average of the 2 evaluators' scores?*

**Added explanation in text. We used the average of values when they differ by up to one degree between the evaluators, discarding evaluations that differed by a higher degree**

*- The distribution of observations in the different categories of fixed effects remains an enigma. How does it vary (min-max)?*

**We added information in the text.**

*- The effects of age class and functionnal class are partially confused. They need to be combined.*

**The model was added to the text. In fact, these effects were combined. We agree that it was not clear.**

*- The rules for choosing genotyped animals are not specified. The genetic structure of the genomic data is essential for assessing the relevance of the results.*

**An analysis with STRUCTURE had been performed in the larger project and was missing from the manuscript. The issue was raised. Information regarding the sampling of animals in terms of grouping clustering was also added.**

*All in all, I recommend major revision to make the attractive conclusions more credible.*

**Considering that not all issues may be resolved, given our doubts and comments, we remain open to further review if necessary.**
